# PEGylated Thermo-Sensitive Bionic Magnetic Core-Shell Structure Molecularly Imprinted Polymers Based on Halloysite Nanotubes for Specific Adsorption and Separation of Bovine Serum Albumin

**DOI:** 10.3390/polym12030536

**Published:** 2020-03-02

**Authors:** Xiufang Li, Hui Liu, Zhiwei Deng, Wenqing Chen, Tianhao Li, Yunshan Zhang, Zhuomin Zhang, Yao He, Zhijian Tan, Shian Zhong

**Affiliations:** 1 College of Chemistry and Chemical Engineering, Central South University, Changsha 410083, China; 2 Institute of Bast Fiber Crops, Chinese Academy of Agricultural Sciences, Changsha 410205, China

**Keywords:** molecularly imprinted polymer, magnetic separation, bovine serum albumin, core-shell structure, specific adsorption

## Abstract

Novel PEGylated thermo-sensitive bionic magnetic core-shell structure molecularly imprinted polymers (PMMIPs) for the specific adsorption and separation of bovine serum albumin (BSA) were obtained via a surface-imprinting technique. X-ray diffraction (XRD), scanning electron microscopy (SEM), transmission electron microscopy (TEM), vibrating sample magnetometry (VSM), fourier transform infrared spectrometry (FT-IR), thermal gravimetric analysis (TGA), and specific surface area (BET), were adopted to demonstrate that novel PMMIPs were successfully synthesized. Subsequently, the prepared PMMIPs were used as the extractor for BSA and were combined with magnetic solid-phase extraction. The concentrations of BSA were detected by UV-vis spectrophotometry at 278 nm. The maximum adsorption capacity of the PMMIPs was 258 mg g^−1^, which is much higher than that of non-imprinted polymer (PMNIPs). PMMIPs showed favorable selectivity for BSA against reference proteins, i.e., bovine hemoglobin, ovalbumin and lysozyme. PMMIPs were further used to recognize BSA in protein mixtures, milk, urine and sewage, these results revealed that approximately 96% of the ideal-state adsorption capacity of PMMIPs for BSA was achieved under complicated conditions. Regeneration and reusability studies demonstrated that adsorption capacity loss of the PMMIPs was not obvious after recycling for four times. Facile synthesis, excellent adsorption property and efficient selectivity for BSA trapping are features that highlight PMMIPs as an attractive candidate for biomacromolecular purification.

## 1. Introduction

Proteins are important biomarkers for certain diseases, health conditions, environmental monitoring, and food quality. The detection of biomarker proteins has become increasingly important, meaning that the construction of biosensors for such biomarker targets has become increasingly important. For the past few years, the molecular imprinting technique (MIT) has received extensive attention in protein separation for its specific identification, efficient selectivity and strong affinity for template molecules [1,2].

Molecularly imprinting is an effective separation method that creates artificial affinity binding sites in polymeric matrices [3]. Molecularly imprinted polymers (MIPs) are synthesized by copolymerization of cross-linkers and functional monomers. Template molecules should be added before the polymerization process and removed afterwards; this allows the functional groups, size, and shape of template molecules to become memorized within the matrix, leaving behind specific recognition cavities [4]. MIPs have advantages of structural predictability and recognition specificity [5]. The chemical stability and selectivity of MIPs are also better than natural receptors [6]. The application of MIPs include purification and separation [7], artificial antibodies [8], target-drug delivery [9,10], and electrochemical sensor [11,12]. Imprinting methods for small molecules (e.g., metal ions [13], pesticide [14] and amino acid [15]) have been well-established, however, the fabrication methods for protein MIPs are still challenging and underdeveloped. Proteins have large sizes and complex structures, and the adsorption and elution of proteins are difficult. Strategies have been developed to overcome these issues, including surface imprinting [16,17], fragment imprinting [18] and epitope imprinting [19,20] during the preparation of protein imprinted polymers.

Many investigators have focused on the combination of surface molecular imprinting techniques with magnetic materials [21,22]. Since imprinting sites are only created on the surface of MIPs, which could provide easy access for template molecules, and be good for the mass transfer and elution. Compared to the traditional support substrates, magnetic materials own excellent properties such as superparamagnetism, hypotoxicity and good biocompatibility. Therefore, when the MIPs covered on the surface of magnetic materials, the synthetic magnetic MIPs can be easily separated from a complicated matrix by using an external magnet; further filtration and centrifugation may be unnecessary.

Since a solid core without an identification site occupies most of the mass of MIPs, it reduces the binding ability of MIPs per unit mass; as is well-known, solid Fe_3_O_4_ microspheres are common support materials in MIT; typically, Gai et al. [23] successfully synthesized BSA surface-imprinted magnetic polymers based on atomic transfer radical polymerization (ATRP) method, and obtained satisfactory selectivity to BSA over the non-imprinted polymer. Chen et al. [24] produced a well-defined imprinted poly-dopamine shell of lysozyme (Lyz) on the surface of Fe_3_O_4_, showing acceptable specific recognition behavior towards Lyz. However, most of the previous published magnetic MIPs got relatively lower recognition capacities owing to the agglomeration of solid Fe_3_O_4_ spheres. Recently, Fe_3_O_4_ microspheres with hollow structures draw more and more attention in many fields [25,26,27]. Through the investigation and comparison of the performance of the hollow Fe_3_O_4_, we infer that the hollow nanotube as the support carrier of imprinting polymer will also have a good effect, meanwhile, the study of magnetic particle modified hollow nanotubes as carrier to imprint proteins is less. Thus, we have begun to towards the use of hollow imprinted nanotubes. Hollow imprinted nanotubes can improve the recognition efficiency between a template molecule and imprinted material. The hollow structure binding site can be fully utilized on the inner and outer surfaces of an MIP via this method. However, traditional non-magnetic hollow imprinted nanotubes are difficult to separate, therefore, column chromatography, centrifugation, or filtration procedures are required for separation purposes [28,29]. Compared with conventional solid support matrixes, halloysite is a natural nanotube mineral with good biocompatibility. It is cheap, rich in reserves, and can also be used as a good carrier for the preparation of protein imprinting materials. Halloysite nanotubes are rich in surface silica groups and aluminum oxygen groups on the inside; both groups are highly modifiable. By using a co-precipitation method to load magnetic particles on the surface and interior of halloysite, and by using magnetic halloysite nanotubes as carriers, imprinted materials with specific recognition and an ability for separation by external magnetic fields can be synthesized; the hollow tubular structure can improve the binding ability of per unit quality in MIPs. 

Thermo-sensitive polymers are “intelligent” materials that can reversibly vary their sizes under outer temperature changes. Magnetic or thermo-sensitive MIPs have been recorded in previous research [30,31]. Notably, these MIPs are beneficial for enhancing mass transfer and recombination efficiency. Among such MIPs, NIPAM has been investigated widely [32,33]. NIPAM has been used peculiarly as a thermo-sensitive gate to regulate the adsorption and elution of biomacromolecule; this is an effective mean that can partly solve problems associated with protein imprinting. However, there were few reports with respect to the combination of all three elements (magnetism, temperature-sensitivity and molecularly imprinting). Gai et al. [23] synthesized BSA surface-imprinted magnetic polymer exhibiting higher adsorption capacity and selectivity to BSA in the presence of common monomer NIPAM, functional monomer *N*-[3-(dimethylamino)propyl]-methacrylamide (DMAPMA) and MAA. Nevertheless, temperature-sensitivity of imprinted polymer was not discussed. In spite of efforts to magnetic or thermo-sensitive MIPs in preceding reports, the adsorption and recognition abilities still lagged behind [32,34].

In this study, we designed and synthesized novel PMMIPs for specific recognition of BSA. PEG-coated, poly (4-Vinylpyridine) modified magnetic halloysite nanotubes (MHNTs@PEG@4-VP) were selected as the substrate material. BSA was acted as template molecules, *N*-isopropylacrylamide (NIPAM), acrylic amide (AAM) and *N*-(3-aminopropyl) methyl acrylamide hydrochloride (APM) as functional co-monomers, and *N,N*-methylene diacrylamide (BIS) as cross-linker. The composition and morphologies of the obtained PMMIPs were characterized using XRD, FT-IR, SEM, TEM, TGA, VSM and BET. The specific adsorption and desorption property of PMMIPs for BSA were further investigated by selective adsorption tests and competitive rebinding experiments. Meaningfully, the practicability of imprinted polymer materials was further evaluated by the isolation of BSA from milk, human urine and sewage.

## 2. Experimental Section

### 2.1. Materials

HNTs were obtained from Zhengzhou Jinyangguang Co. Ltd. (Henan, China). The 4-vinylpyridine (4-VP), ethylene glycol dimethacrylate (EGDMA), 2,2′-azobis(2-methylpropionitrile) (AIBN), ferric (III) salts (FeCl_3_.6H_2_O), ferrous(II) salts (FeCl_2_.4H_2_O), *N*-isopropyl acrylamide (NIPAM), acrylic amide (AAM), *N,N*-methylene diacrylamide (BIS), *N,N,N′,N′*-tetramethyl ethylenediamine (TEMED), polyethylene glycol 6000 (PEG 6000), ammonium hydroxide, and N-(3-aminopropyl) methyl acrylamide hydrochloride (APM) were purchased from Aladdin Chemical Company (Shanghai, China). Bovine serum albumin (BSA, *M*_w_ = 68 kDa, pI = 4.9), ovalbumin (OVA, *M*_w_ = 43 kDa, pI = 4.7), lysozyme (Lyz, *M*_w_ = 14.4 kDa, pI = 11.2), bovine hemoglobin (BHb, *M*_w_ = 64.5 kDa, pI = 6.9) were obtained from Micxy Reagent Co. Ltd. (Chengdu, China). Ammonium persulphate (APS), sodium dodecyl sulfate (SDS), glacial acetic acid were obtained from Sinopharm Chemical Reagent Co., Ltd (Shanghai, China). All chemicals were of analytical reagent grade, all water was doubly distilled before use. 

### 2.2. Preparation of PMMIPs and PMNIPs

New type of PMMIP was synthesized via combination of precipitation polymerization and surface imprinting technique. First, MHNTs@PEG and MHNTs@PEG@4-VP were prepared, the synthetic methods of them were described in the Supporting Information. Next, the as-prepared MHNTs@PEG@4-VP were dispersed into phosphate buffer (PB) solution (0.1 mol L^−1^, pH = 7.0, 30.0 mL) by ultrasonic vibration for 30 min. The template protein BSA was then added into the mixture and dissolved by vibration. Then NIPAM, AAM and APM were dissolved in the above mixture under argon atmosphere. The solution was magnetically stirred for 1 h at room temperature. The crosslinking agent BIS was injected into the reaction mixture using a syringe. The solution was gently magnetically stirred for prepolymerization at 35 °C for 5 h and subsequently deoxygenated by blowing argon. Next, initiator APS (100.0 mg dissolved into 5.0 mL PB solution) and catalyst TEMED (50.0 µL) were consecutively injected into above solution. The reaction proceeded under vigorous stirring at 40 °C for 24 h. The PMMIPs were obtained by external magnetic separation and were washed with deionized water to remove redundant functional monomer and attached oligomers. Then the template protein was eluted using SDS (10%, *w*/*v*)-acetic acid (10%, *v*/*v*) at 25 °C until no BSA was detected in the supernatant by UV-Vis absorption (Shimadzu, Japan). PMMIPs were obtained by external magnetic separation after washed with deionized water and vacuum dried at 30 °C for 12 h. PMNIPs were obtained as control by using the same method but without the template BSA.

### 2.3. Adsorption Experiments

Adsorption tests contained the adsorption isotherms and adsorption kinetics. All adsorption tests were implemented in screw thread bottles. The batch technique was adopted to investigate the effect of experimental parameters, including adsorbed time and the initial concentration of BSA solution. The concentration of BSA in the supernatant was analyzed employing a UV-vis spectrophotometer at 278 nm after being filtered via a Millipore cellulose nitrate membrane with a pore size of 0.45 μm.

#### 2.3.1. Adsorption Isotherms 

The adsorption properties of PMMIPs and PMNIPs were surveyed by static adsorption tests. Briefly, 10.0 mg PMMIPs or PMNIPs were individually dispersed into 10.0 mL of PBS containing various concentrations of BSA. The initial concentration range of BSA solution was from 0.2–2.0 mg mL^−1^, the work temperature was set at 20 °C and vibrated for 5 h. After magnetic separation and filtration, the concentration of the supernatant was analyzed utilizing a UV-Vis spectrophotometer. The amount of protein adsorbed on the polymers *Q*_e_ (equilibrium adsorption capacity, mg g^−1^) was calculated from the protein concentrations before and after adsorption using the following equation:

$$Q_{e}=\left(C_{o}\;-{\;C}_{e\;}\right)V/m$$

where *C*_o_ and *C*_e_ represent the initial and the equilibrium BSA concentrations in the solution (mg mL^−1^), respectively; V (mL) is the volume of the solution; and m (g) is the amount of PMMIPs and PMNIPs, respectively.

#### 2.3.2. Adsorption Kinetics 

Adsorption kinetics is vital for assessing the adsorption efficiency of PMMIPs (PMNIPs). To discovery the adsorption process, 10.0 mg of PMMIPs or PMNIPs were dispersed into 10.0 mL of BSA solution at the initial concentration of 1.0 mg mL^−1^, and were investigated by varying the adsorption time from 10–180 min. PMMIPs or PMNIPs were then separated using an external magnet from the solution. The concentration of BSA in the supernatant was measured by UV-Vis spectrometry at 278 nm. The adsorbed amount *Q*_e_ was obtained according to measuring the BSA concentration before and after adsorption using UV-vis spectrometer. Calculation formula for *Q_e_* (mg g^−1^) is the same as Equation (1).

### 2.4. Adsorption Selectivity

Studies were carried out to evaluate the specific rebinding of PMMIPs to BSA; three other proteins (BHb, OVA and Lyz) with different pIs and Mw were selected as reference proteins. In the selective adsorption experiments, PMMIPs or PMNIPs (10.0 mg) were added to 10.0 mL solution of deionized water with 1.0 mg mL^−1^ of single component (BSA, BHb, OVA or Lyz), respectively, and the mixtures were shaken in a thermostatic shaker for 5 h at 20 °C. After magnetic separation and filtration through a 0.45 µm filtration membrane, the supernatant concentration was measured by ultraviolet absorption spectrophotometry. The UV-vis absorption wavelengths of BSA, OVA and Lyz were 278 nm, and BHb was 405 nm. The adsorbed amount of BSA by the PMMIPs was measured according to the previously described equation. 

### 2.5. Removal of BSA from Real Samples

To verify the effectiveness of PMMIPs for use in real samples, milk, urine and sewage were used in place of deionized water solution. Milk was pretreated before use to remove protein particles by adding 10.0 mL methanol into 2.0 mL milk followed by centrifugation. Prior to use, milk, urine and sewage were tested with a UV-vis spectrometer at 278 nm and no BSA was detected. Experiments were carried out with similar procedures as the adsorption isotherm experiments with BSA concentration of 1.0 mg mL^−1^. Meanwhile, competitive adsorption tests were also conducted in real samples, BHb was selected as reference protein, the experiments were carried out with similar procedures as adsorption isotherm experiments with the BSA and BHb concentration of 1.0 mg mL^−1^, respectively.

### 2.6. Regeneration and Reproducibility

Reproducibility and regeneration are one of the important features for the recycling of an imprinted material. The regeneration and reproducibility of PMMIPs and PMNIPs were estimated by executing four successive cycles of adsorption-desorption of the same test sample. Briefly, PMMIPs or PMNIPs (10.0 mg) were mixed with BSA/PBS at an initial concentration of 1.0 mg mL^−1^ and shaken on a table concentrator at 20 °C for 5 h. After specific adsorption, the samples were washed with SDS (10%, *w*/*v*) and acetic acid (10%, *v*/*v*) solution to remove BSA; BSA was then rebound to the samples.

## 3. Results and Discussion

### 3.1. Preparation of PMMIPs

**Scheme 1:**
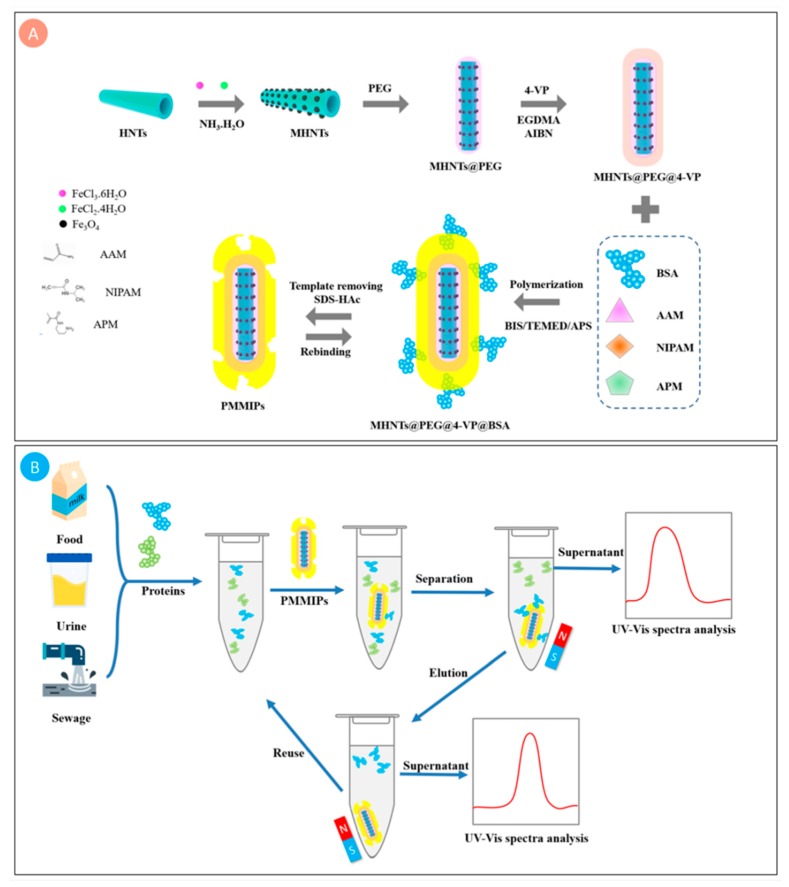
Schematic illustration of (**A**) PMMIPs synthesis procedure; (**B**) adsorption and separation of BSA from a mixture of proteins using PMMIPs. PMMIPs, novel PEGylated thermo-sensitive bionic magnetic core-shell structure molecularly imprinted polymers.

The overall synthetic route of PMMIPs and our protein captured and released strategy are drawn in Scheme 1. Firstly, MHNTs@PEG were synthesized by adopting an improved one-pot co-precipitation method, which was simpler than other steps methods. PEG was served as a hydrophilic network to increase the dispersion and stability of the material; the second step was to graft a double-bond compound 4-VP on the surface of MHNTs@PEG through polymerization. The third step was to form MIPs layer while BSA served as template. NIPAM, AAM and APM as functional co-monomers, among NIPAM acted as a thermo-sensitive monomer to realize expansion and shrinkage of PMMIPs. The adsorption and elution of BSA were achieved in answer to the temperature changes. Therefore, the ability of PMMIPs to capture and release BSA could be controlled by changing the outer temperature. More significantly, the poly (4-VP) layer not only hinders Fe_3_O_4_ oxidation, but enhances chemical stability. Pyridyl is a hydrophilic group, which could enhance the dispersion of PMMIPs in aqueous solution. Furthermore, the pyridyl group and imprinted polymer layers could form hydrogen bonds with protein, which could facilitate selective adsorption of BSA. After removing the template protein, PMMIPs were obtained which owned imprinted sites complementary to the template in shape, dimension, and functional group orientation.

### 3.2. Characterization of PMMIPs

#### 3.2.1. Composition and Morphological Characterization

SEM and TEM tests were used to investigate the morphological features and dimensions of the prepared magnetic imprinted materials. As shown in Figure S1a (Supplementary Materials), MHNTs@PEG resembled a rod-shape, there are many magnetic nanoparticles on it. Comparing with MHNTs@PEG, the surface of MHNTs@PEG@4-VP (Figure S1b in Supplementary Materials) had become smooth and the pore spacing was smaller, which due to the nanotubes was covered by 4-VP polymer. As shown in Figure S1c,d (Supplementary Materials), compared with MHNTs@PEG@4-VP, PMMIPs and PMNIPs had dents on their surfaces, respectively; this was because the PMMIP shell produced an imprinting cavity after BSA was removed by 10% (*w*/*v*) SDS-10% (*v*/*v*) acetic acid; however, the imprinting cavity of PMNIPs shell layer was not obviously due to the lack of template molecules. MHNTs@PEG exhibited hollow tubular structures with a ~50 nm external diameter, ~20 nm inner diameter, ~400 nm length, and 25 nm wall thickness. Furthermore, the Fe_3_O_4_ agglomerated on the inside and outside of hollow HNTs, (Figure S1e in Supplementary Materials). Comparing with MHNTs@PEG, the TEM of MHNTs@PEG@4-VP (Figure S1f in Supplementary Materials) showed that the surface of nanotubes become smooth due to the coating of the polymer layer, resulting in less exposure of magnetic nanoparticles, the thickness of the MHNTs@PEG@4-VP layer was 25~28 nm. As shown in Figure S1g (Supplementary Materials), the outer surface of MHNTs@PEG@4-VP appeared much smoother after modification and polymerization; the thickness of PMMIPs layer was 25–30 nm, which was further evidence that PMMIPs grew by selectively grafting on the exterior of MHNTs@PEG@4-VP; However, the imprinted layer thickness of PMNIPs (Figure S1h in Supplementary Materials) was a little thin due to the lack of BSA. More significantly, the results of SEM micrographs and TEM images provided strong evidence for the successful synthesis of PMMIPs.

#### 3.2.2. FT-IR Spectra

**Figure 1:**
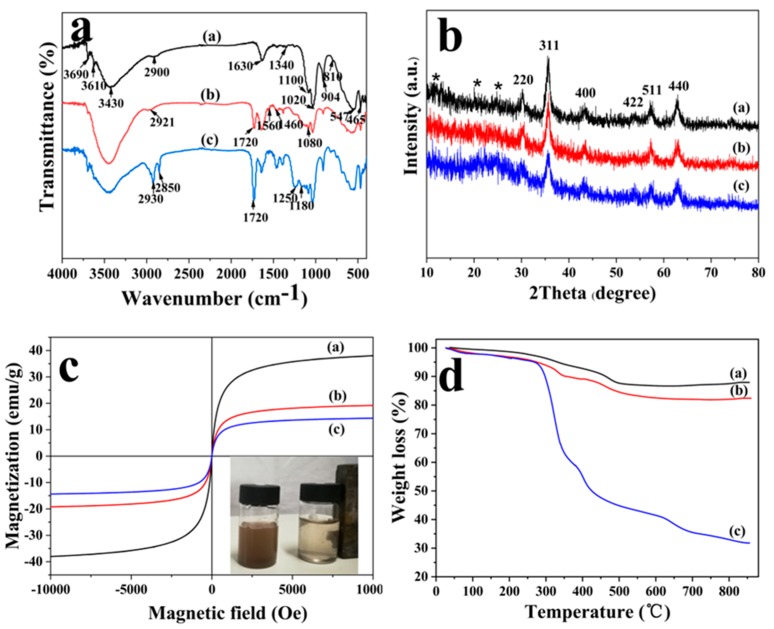
(**a**) FT-IR spectra of (a) MHNTs@PEG, (b) MHNTs@PEG@4-VP, (c) PMMIPs; (**b**) XRD pattern of (a) MHNTs@PEG, (b) MHNTs@PEG@4-VP, (c) PMMIPs; (**c**) VSM curves of (a) MHNTs@PEG, (b) MHNTs@PEG@4-VP, (c) PMMIPs; (**d**) TGA plots of (a) MHNTs@PEG, (b) MHNTs@PEG@4-VP, (c) PMMIPs.

The FT-IR of MHNTs@PEG, MHNTs@PEG@4-VP and PMMIPs were displayed in Figure 1. The peaks at 3690 and 3610 cm^−1^ were attributed to the stretching vibrations of the inner-surface Al-OH groups based on the FT-IR spectrum of PMHNTs (Figure 1a(a)). Interlayer water was indicated by bending vibration at 1630 cm^−1^. The peaks at 1020 and 1100 cm^−1^ were assigned to the stretching mode of apical Si–O–Si. The peak at 904 cm^−1^ was attributed to the bending vibration of the inner-surface hydroxyl groups. The peaks at 547, 465, 810, 1340, 2900 and 3430 cm^−1^ were attributed to vibration Fe–O of Fe_3_O_4_, C–O–C, C–C, –CH_2_– and O–H of PEG, that was strong evidence for the existence of Fe_3_O_4_ and PEG. As shown in Figure 1a(b), a new peak appeared at 1720 cm^−1^, the absorption intensity increased within the range of 1080 to 1460 cm^−1^ and the peaks at 2921 and 1560 cm^−1^ were assigned to the –CH_2_– group and pyridine group, respectively, indicating that the graft of poly(4-VP) layer was successful. Additionally, new adsorption bands appeared at 2930 and 2850 cm^−1^, which were attributed to –CH_2_– group; the increase of adsorption intensity at 1720 cm^−1^ and the appearance of new peaks at 1250 cm^−1^ in Figure 1a(c) were ascribed to C=O of NIPAM, AAM and APM. These data illustrated that MIPs were successfully grafted onto the surface of the support MHNTs@PEG.

#### 3.2.3. X-ray Diffraction

XRD characterization was implemented for series of MHNTs compounds, Figure 1b displayed the XRD modes of MHNTs@PEG, MHNTs@PEG@4-VP composite, and PMMIPs. Based on the XRD pattern, the inverse cubic spinel structure of Fe_3_O_4_ was determined. The main peaks of all products were at 2θ = 30.2° (220), 35.5° (311), 43.2° (400), 53.5° (422), 57.2° (511) and 62.4° (440), which were consistent with the characteristic peaks of Fe_3_O_4_ (JCPDS 19-0629). The broad diffraction peaks at 2θ = 12°, 21°, and 25° in MHNTs@PEG, MHNTs@PEG@4-VP and PMMIPs, originated from the raw HNTs. Comparing with the existing models, there were no new crystals produced after decoration with MIPs. After successfully modifying with polymers, the peak strength of MHNTs@PEG@4-VP and PMMIPs were slightly reduced due to polymers covering the imprinted shell on the MHNTs@PEG. These results indicated the presence of Fe_3_O_4_ in MHNTs@PEG, MHNTs@PEG@4-VP and PMMIPs.

#### 3.2.4. Magnetic Properties

Magnetism is a vital property of PMMIPs, which can ensure magnetic MIPs could quickly be separated from the medium. Magnetic features of PMMIPs were executed by vibrating sample magnetometer at room temperature. As shown in Figure 1c, the saturated magnetic intensities of MHNTs@PEG, MHNTs@PEG@4-VP and PMMIPs were 38.05, 19.19 and 14.37 emu/g, respectively. The magnetism of PMMIPs was strong enough to achieve magnetic separation, though it marginally decreased relative to that of MHNTs@PEG@4-VP. The decline of magnetization was attributed to the nonmagnetic polymer layers that covered on the surface of MHNTs@PEG, which coincided well with the above-mentioned TEM conclusions.

#### 3.2.5. TGA and DTG Analysis

To further study the grafting of MIPs on the MHNTs@PEG, TGA was implemented to quantize the weight component of MIPs. The TGA results for MHNTs@PEG, MHNTs@PEG@4-VP and PMMIPs were shown in Figure 1d. The total weight loss of MHNTs@PEG was approximately 12.06%, which was likely due to the loss of water and PEG, and that of MHNTs@PEG@4-VP was approximately 17.60%, which was likely ascribe to the loss of water, PEG, and 4-VP; that of PMMIPs was approximately 68.19%, which was likely ascribe to the loss of water, PEG, 4-VP, and MIPs. Comparing the three data, the relative weight of MIPs in PMMIPs nanomaterial was approximately 50.59% and the weight of 4-VP in the MHNTs@PEG@4-VP nanomaterial was approximately 5.54%. The results confirmed that MIPs and 4-VP were assuredly grafted onto MHNTs@PEG, which coincided well with FT-IR results. 

To further investigate the thermal decomposition of PMMIPs, we had also performed DTG analysis. As could be seen from the DTG curve (Figure S2 in Supplementary Materials), MHNTs@PEG (Figure S2a) had no weight loss between 50–280 °C, a small amount of weight loss began to appear between 280–350 °C, which may be due to the partial decomposition of the compound modified on the nanotubes; then, the pyrolysis reaction tended to be gentle, and the sample quality was not reduced, it appeared as a straight line on DTG curve between 350–450 °C; and then it entered the most violent stage of thermal reaction between 450–550 °C, DTG curve decreased significantly, it belonged to the stage of rapid weightlessness, which mainly due to the complete decomposition of the polymer on the nanotubes. When the temperature was higher than 550 °C, the pyrolysis reaction was basically smooth and the sample quality was not reduced, it appeared as a straight line on DTG curve, it showed that the pyrolysis reaction was basically completed. Meanwhile, the thermal decomposition process of MHNTs@PEG@4-VP (Figure S2b) was similar to that of MHNTs@PEG, the difference between them was that MHNTs@PEG@4-VP showed obvious weight loss between 280–350 °C. With the increase of polymer layer thickness, thermal decomposition of PMMIPs (Figure S2c) was becoming more and more obvious, the pyrolysis reaction was the most intense in the range of 280–380 °C, a sharp peak appeared in the DTG curve. At the same time, the weight loss rate of PMMIPs between 380–450 °C was also obvious compared with MHNTs@PEG and MHNTs@PEG@4-VP, a new peak of DTG also appeared at 580–650 °C, which due to the formation of a new imprinted layer outside the nanotubes. When the temperature was higher than 700 °C, the pyrolysis reaction tended to be gentle, and the sample quality was not reduced, it appeared as a straight line on DTG curve, it showed that the pyrolysis reaction was basically completed. This result was consistent with the TGA of PMMIPs.

#### 3.2.6. BET Analysis

**Figure 2:**
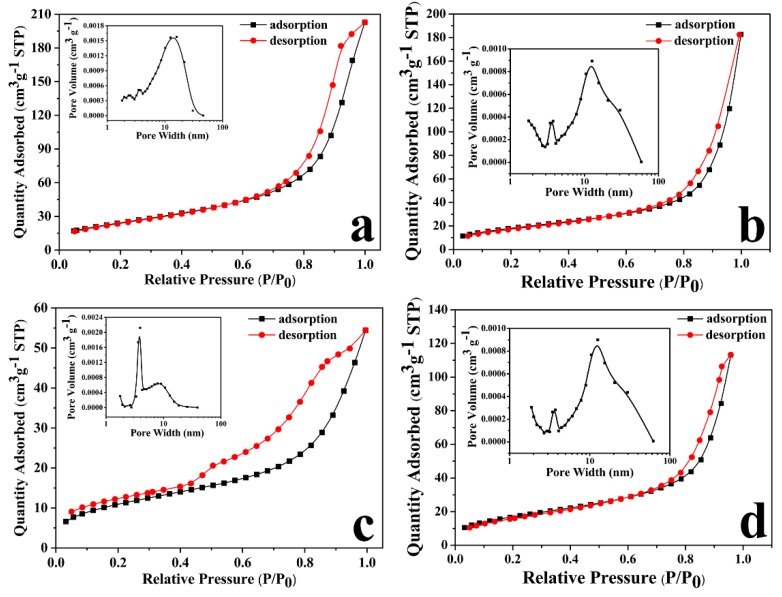
N_2_ adsorption-desorption isotherm of (**a**) MHNTs@PEG, (**b**) MHNTs@PEG@4-VP, (**c**) PMMIPs and (**d**) PMNIPs with BET; (inset) pore size distribution of (**a**) MHNTs@PEG, (**b**) MHNTs@PEG@4-VP, (**c**) PMMIPs and (**d**) PMNIPs.

Surface area analysis was carried out on a series of MHNTs compounds by the BET method. The N_2_ adsorption/desorption isotherm of MHNTs@PEG, MHNTs@PEG@4-VP, PMMIPs and PMNIPs were typical type-IV with BET of 90.30, 66.55, 39. 95 and 60.47 m^2^ g^−1^ (Figure 2), respectively. In the low-pressure region (P/P_0_ < 0.8), the adsorption isotherms were relatively flat, while in the high relative pressure region (P/P_0_ > 0.8), the isotherms increased rapidly. The BJH pore size distribution curve of the MHNTs@PEG was shown in Figure 2a. The pores ranged from 2 to 60 nm, which indicated that mesopores and macropores coexisted on MHNTs@PEG. The peaks around 12, 5 and 3 nm were attributed to the nanotube lumen, pores among the tubes and surface defects, respectively. After modification with 4-VP, there appeared mesopores in MHNTs@PEG@4-VP, the pore diameter distributed between 3 and 13 nm (Figure 2b); and after modification with MIPs, there appeared mesopores in PMMIPs, the pore diameter distributed between 5 and 12 nm (Figure 2c), meanwhile, the pore diameter of PMNIPs without template distributed between 3 and 12 nm (Figure 2d). Furthermore, the BET of PMMIPs decreased to 39.95 m^2^ g^−1^. The decline of BET and pore volume of the modified MHNTs@PEG was mainly owing to PEG, 4-VP and MIPs coating which could blocke the pores. Meanwhile, the BET of PMNIPs decreased to 60.47 m^2^ g^−1^, which due to the fact that MIPs were affected by the absence of template molecules.

#### 3.2.7. Experiments to Optimize the Reaction Parameters of MIPs Based on the MHNTs@PEG@4-VP Nanotubes

In order to screen out the optimal obtained conditions of MIPs based on the 4-VP-modified MHNTs@PEG nanotubes, the influences of template: BSA, functional monomers: NIPAm, AAM, APM and cross-linkering agent: Bis on *Q*_e_ and *IF* of PMMIPs were investigated. The synthetic parameters and obtained results were shown in Table S1. Primarily, the influence of additive amount of BSA was studied (MIPs 1–5), the experiment data indicated that *Q*_e_ increased with the addition of template increased due to the more binding sites was. When the superfluous template was added, functional monomer would be insufficient to combine all the templates and mold the matching spatial structure of MIPs, therefore, the *Q*_e_ was weaken as the *IF* value gone down (MIPs 5). Next, the influence of functional monomers (NIPAm, AAM, APM) for *Q*_e_ was studied (MIPs 4, 6–9), the data exhibited that *Q*_e_ and *IF* of PMMIPs increased with the amount of monomers when the moles from 1 to 4 mmol, displaying that the increase of monomers would be beneficial to the formation of recognition sites. Whereas, superfluous addition of the monomers would lead to decrease of *Q*_e_ and *IF* of PMMIPs since the MIPs layer would be too thick to transfer BSA and owned more nonspecific adsorption sites (MIPs 9). Finally, the influence of crosslinking agent (BIS) was also studied (MIPs 8, 10–12). *Q*_e_ and *IF* increased with the amount of BIS from 0.5 to 1 mmol, owing to the crosslinking agent could reinforce the structure of recognition cavities in MIPs. However, *Q*_e_ and *IF* decreased since the amount of BIS was >1 mmol, because the superabundant crosslinking agents would impede protein transport. In brief, the optimal experimental conditions were as follows: template: BSA (50 mg), functional monomers: NIPAm, AAM and APM (4 mmol) and crosslinking agent: BIS (1 mmol), respectively.

#### 3.2.8. Thermo-Sensitivity Analysis

**Figure 3:**
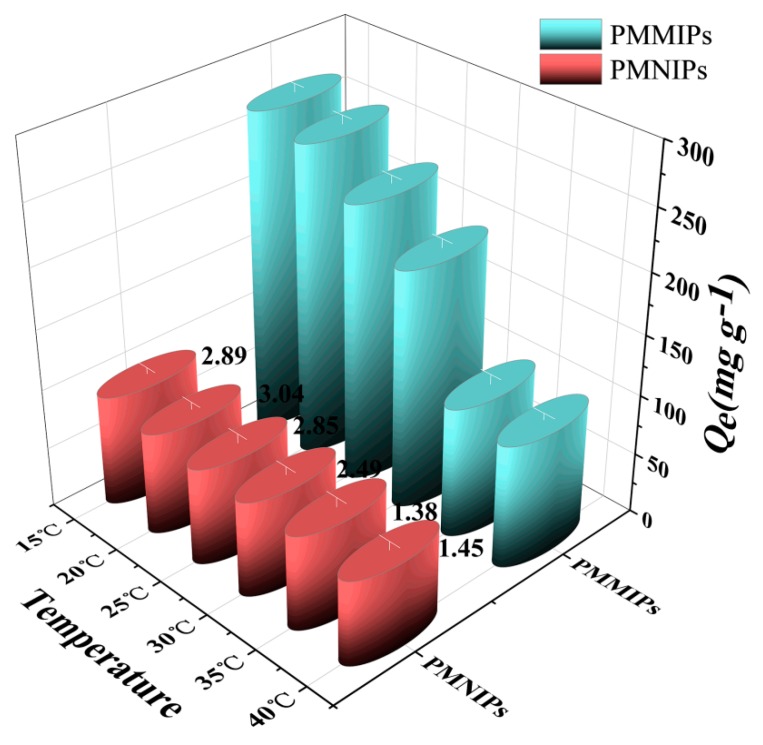
The adsorption capacity of PMMIPs and PMNIPs for BSA at different temperatures. (Adsorption conditions: V = 1 mL, m_PMMIPs_ = m_PMNIPs_ = 10.0 mg, C_BSA_ = 1.0 mg mL^−1^, T = 15–40 °C, t = 5 h, pH = 7.0).

The adsorption capacity of PMMIPs or PMNIPs for BSA was investigated at temperatures from 15 to 40 °C (Figure 3). It could be concluded that *Q*_e_ of PMMIPs or PMNIPs for BSA slightly decreased as temperature increased. However, the *Q*_e_ of PMMIPs decreased more rapidly than that of PMNIPs. Peculiarly, the *Q*_e_ dropped sharply when the temperature had gone from 30 to 35 °C, but it had gone down very slowly from 35 to 40 °C. The explanation for this result was as follows: the matrix network of the MIPs layer would shrink with the increase of temperature, which would hinder BSA from entering the polymer matrix in a shrinkage state, besides, the morphology and size of imprinting hole and spatial distribution of functional groups in the MIPs layer of PMMIPs would change. Hence, MIPs were unable to match its templates and the affinity dramatically decreased [35,36]. When the temperature was above the lower critical solution temperature (LCST is about 32 °C) of NIPAM, the MIPs layer would severely collapse, leading to lower affinity [37,38]. The *Q*_e_ of the PMNIPs decreased more slowly with the temperature, because the PMNIPs had no template imprinting layer. In short, PMMIPs owned the best imprinting effect of BSA at 20 °C by reason that the highest imprinting factor (*IF* = 3.04). The desorption capacity of PMMIPs (PMNIPs) for BSA at 35 °C was also further studied via their thermo-sensitivity performances; when elution was executed once, BSA desorbed considerably. This was due to the weakening in imprinted cavities effects when the elution temperature was above LCST, which would promote template molecule escape from the imprinted cavities. These results indicated that the PMMIPs (PMNIPs) displayed excellent thermo-sensitive, which was significant for absorption and desorption of template molecule.

### 3.3. Adsorption Property

#### 3.3.1. Adsorption Isotherms

**Figure 4:**
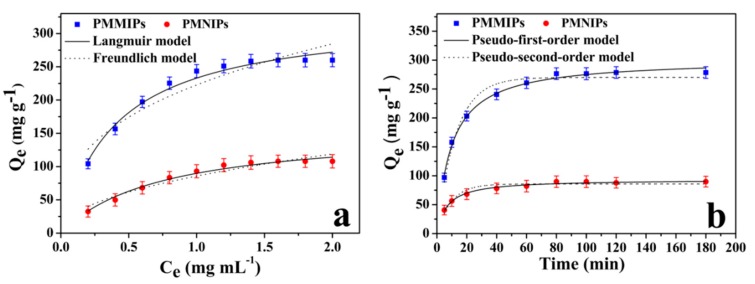
(**a**) Adsorption isotherm curves of BSA on PMMIPs and PMNIPs. Adsorption conditions: V = 1 mL, m_PMMIPs_ =m_PMNIPs_ = 10 mg, C_BSA_ = 0.2– 2.0 mg mL^−1^, T = 20 °C, t = 5 h, pH = 7.0; (**b**) adsorption kinetics curves of PMMIPs and PMNIPs. Adsorption conditions: V = 1 mL, m_PMMIPs_ = m_PMNIPs_ = 10.0 mg, C_BSA_ = 1.0 mg mL^−1^, T = 20 °C, t = 3 h, pH = 7.0.

Figure 4a presented the adsorption isotherms of PMMIPs and PMNIPs for BSA. The amount of BSA which was adsorbed by both PMMIPs and PMNIPs increased when the initial BSA concentration was increased from 0.2 to 1.4 mg mL^−1^, when the BSA concentration reached 1.4 mg mL^−1^, the adsorption capacity reached equilibrium (258.4 mg g^−1^), which was higher than those of the other MIPs particles reported [32,39,40], and the adsorption capacity of BSA onto PMNIPs was 106.1 mg g^−1^. Consequently, *IF* was 2.44 by calculation, which was higher than those of the other MIPs particles reported [23,41,42,43]; notably, the PMMIPs displayed a higher binding capacity than PMNIPs. The major difference between PMMIPs and PMNIPs was that PMMIPs owned specific recognition cavities, while PMNIPs lacked relevant imprinting cavities due to no template molecule. Hence, this result could be ascribed to porous structure of PMMIPs which matched template BSA chemically and spatially.

Langmuir and Freundlich isotherm models were used to further study the adsorption isotherm of BSA on PMMIPs and PMNIPs. The Langmuir isotherm presupposes that adsorption behavior is based on mono-layer adsorption, which assumes that each recognition site adsorbs only one target molecule and the adsorption energy is equal at each recognition site; in addition, that there are no interactions between target molecules. However, the Freundlich model is a multilayer adsorption model, which proposes that adsorption takes place on the heterogeneous surface, and that affinity and adsorption energy of adsorption materials to target molecules are determined by specific surface properties. The two models are expressed by the following formulas, respectively:

$$\mathrm{Langmuir}\;\mathrm{model}:\;Q_{e}=\frac{K_{L}Q_{m}C_{e}}{{1+K}_{L}C_{e}}$$

$$\mathrm{Freundlich}\;\mathrm{model}:\;Q_{e}{=K}_{F}C_{e}^{\frac{1}{n}}$$

where *Q*_e_ (mg g^−1^) is equilibrium adsorption capacity, *C*_e_ (mg L^−1^) represents equilibrium concentration of BSA during adsorption, *K*_L_ (L mg^−1^) is the Langmuir adsorption equilibrium constant, *Q*_m_ (mg g^−1^) is the maximum adsorption capacity of PMMIPs or PMNIPs, *K*_F_ (mg g^−1^) is the Freundlich adsorption equilibrium constant, and 1/*n* is a factor used to evaluate the reaction intensity or surface heterogeneity. A value of 1/*n* smaller than 1.0 represents a favorable removal condition.

The parameters of nonlinear fitting results of these two models are illustrated in Table S2 (Supplementary Materials). Based on R^2^ (correlation coefficients) values, the Langmuir model fits the data better than Freundlich model for PMMIPs and PMNIPs. This suggests that monolayer adsorption may exist between target molecules BSA and PMMIPs or PMNIPs.

#### 3.3.2. Adsorption Kinetics 

The adsorption kinetics of the PMMIPs (PMNIPs) for BSA were studied by time-dependent studies (Figure 4b). Adsorption kinetics are significant way to evaluate the adsorption efficiency of PMMIPs and PMNIPs. Kinetic adsorption tests were carried out in PBS buffer (pH = 7.0) with 1.0 mg mL^-1^ BSA. During the first 40 min, the adsorption kinetics of PMMIP rapidly increased due to numerous empty binding sites on the surface. As time increased, the adsorption rate of PMMIPs declined, and reached equilibrium within 80 min; The equilibrium time of the PMMIPs in this work was shorter than that of some other surface imprinting technologies for BSA [27,34,44,45]; 87% of the rebinding amount was achieved within 40 min, followed by a slow increase over time. Explanations for this phenomenon that the most binding sites were occupied, and the concentration of BSA had decreased. Additionally, the molecularly imprinted cavities may have been fully occupied, and adsorption may have reached equilibrium. This indicated that a large proportion of the imprinted sites were indeed located on the surface of the polymeric matrix. The kinetic rebinding of BSA to PMNIPs was significantly slower when compared with that of BSA to PMMIPs, which could be ascribed to nonspecific adsorption. These satisfactory performances suggested that surface imprinting technology can encourage imprinted sites to distribute on the external surface of PMMIPs. Hence, PMMIPs containing surface-exposed imprinted sites allowed efficient analyte diffusion to imprinted sites, thus bringing about fast kinetic rebinding.

In order to further analyze the results, two types of adsorption models were introduced: a pseudo-first-order model and a pseudo-second-order model [46,47]. They are defined as follows:

$$\mathrm{Pseudo}-\mathrm{first}-\mathrm{order}\;\mathrm{model}:\;Q_{t}{=Q}_{e}{\;-\;Q}_{e}e^{{-k}_{1}t}$$

$$\mathrm{Pseudo}-\sec\mathrm{ond}-\mathrm{order}\;\mathrm{model}:\;Q_{t}\;=\;\frac{k_{2}Q_{e}^{2}t}{{1+k}_{2}Q_{e}t}$$

where *Q*_t_ (mg g^−1^) is the adsorption capacity at adsorption time *t* (min), *Q*_e_ (mg g^−1^) is equilibrium adsorption capacity, and *k*_1_ (mg g^−1^) and *k*_2_ ((g/(mg min)) represent the adsorption rate constant of the pseudo-first-order model and pseudo-second-order model, respectively.

As illustrated in the Table S3, the pseudo-first-order model fits the data better to PMMIPs and PMNIPs than the pseudo-second-order model, based on the R^2^ value. This suggests that physical interactions may exist between adsorbents and BSA, and that the adsorption process may be controlled by the movement of template molecules in PMMIPs.

### 3.4. Adsorption Selectivity

**Figure 5:**
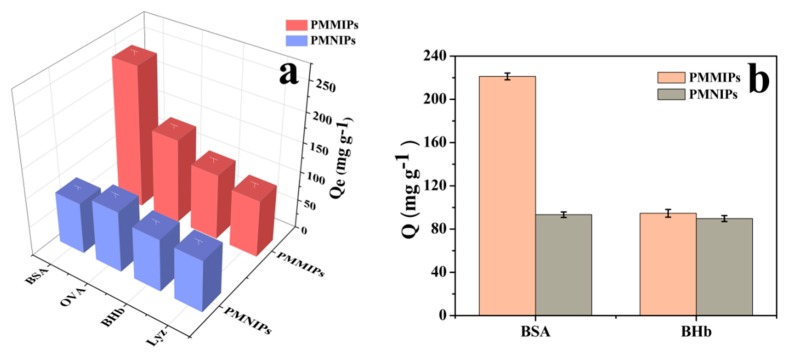
(**a**) Adsorption capacity of PMMIPs and PMNIPs for different protein molecules. Adsorption conditions: V = 1 mL, m_PMMIPs_ = m_PMNIPs_ = 10.0 mg, C_0_ = 1.0 mg mL^−1^, T = 20 °C, t = 5 h, pH = 7.0. (**b**) Competitive adsorption tests of BSA and BHb on PMMIPs and PMNIPs. Adsorption conditions: V = 1 mL, m_PMMIPs_ = m_PMNIPs_ = 10.0 mg, C_BSA_ = C_BHb_ =1.0 mg mL^−1^, T=20 °C, t = 5 h, pH = 7.0.

To investigate the binding specificity of PMMIPs, a selectivity test was implemented with BSA, BHb, OVA and Lyz. These proteins have different pI and *M*_w_. Compared with template BSA, BHb has a similar MW but different pI. Both OVA and Lyz have smaller sizes and different pIs to BHb; the pI of Lyz is 11.2 (>7) while the pI of OVA is 4.7 (<7). The adsorption capacity results of PMMIPs and PMNIPs for different proteins are illustrated in Figure 5a. The adsorption capacities of PMNIPs were not significantly different because selective recognition sites are absent in PMNIPs. PMMIPs exhibited excellent selectivity for BSA relative to other proteins. The *Q*_e_ of PMMIPs for BSA, OVA, BHb and Lyz were 243.50, 145.60, 113.40 and 98.6 mg g^−1^, respectively. These results indicated that PMMIPs have superior specific recognition for BSA. The excellent selectivity for BSA may be due to specific recognition sites that match with the template BSA in size, shape and the placement of functional groups. OVA and BSA have similar pI, and OVA’s *M*_w_ is slightly smaller than BSA; however, OVA has a different shape to BSA, therefore, OVA may not be easy to enter the BSA imprinted cavity. 

The size of Lyz is smaller and Lyz have different pI with BSA. At pH 7.0, BSA has a negative charge and Lyz has a positive charge, thereby reducing the adsorption of Lyz on the two polymer materials. BHb is almost the same size as BSA, however, the adsorption capacity of PMMIPs for BHb was much lower than that for BSA, which once again testified that a shape-memory effect played a dominant role in forming an imprint and in template recognition.

To quantitatively describe the result of the adsorption selectivity experiment, several parameters were introduced, namely, the distribution coefficients (*K*_d_), selectivity coefficients (*k*), and relative selectivity coefficient (*k*′) [48] of BSA, OVA, BHb and Lyz. Their equations are listed as follows:

$$K_{d}=\frac{Q_{e}}{C_{e}}$$

where *K*_d_ (L g^−1^) means the distribution coefficient, *Q_e_* (mg g^−1^) is the equilibrium adsorption capacity of PMMIPs or PMNIPs, and *C_e_* (mg L^−1^) represents the equilibrium concentration of PMMIPs or PMNIPs. According to the *K*_d_ value, the selectivity coefficients (*k*) of PMMIPs or PMNIPs for BSA, OVA, BHb and Lyz can be calculated:

$$k=\frac{K_{d}\left(\mathrm{BSA}\right)}{K_{d}\left(R\right)}$$

where *k* is the selectivity coefficient, and R represents reference molecules (OVA, BHb and Lyz). The relative selectivity coefficient (*k*′) can be calculated according to the *k* of reference molecules, which is defined as:

$$k^{\prime }=\frac{k_{M}}{k_{N}}$$

where *k*_M_ is the selectivity coefficient of PMMIPs, and *k*_N_ is the selectivity coefficient of PMNIPs.

Table S4 (Supplementary Materials) shows the results of *K*_d_, *k*, and *k*′. *K*_d_ is an indicator of the adsorption capacity of PMMIPs or PMNIPs for BSA, OVA, BHb and Lyz k represents the adsorption selectivity of PMMIPs or PMNIPs for template molecules. The larger the k value is, the better the selectivity effect of PMMIPs or PMNIPs for the template molecule is *k′* represents the adsorption affinity of recognition sites of PMMIPs or PMNIPs for template molecules. The larger the *k*′ value is, the better the effect of imprinting is.

The *K*_d_ values of PMMIPs for BSA, OVA, BHb and Lyz were 1.16, 0.45, 0.33 and 0.27, respectively. This revealed that the adsorption capacity of PMMIPs for BSA was much greater than that for OVA, BHb or Lyz. However, no significant differences were found between the *K*_d_ value of PMNIPs for BSA and that for OVA, BHb or Lyz (0.32 versus 0.49, 0.34 and 0.28, respectively). This demonstrated that the affinities of PMNIPs for the three molecules were similar and hence there was a lack of selectivity. It could be seen more clearly that, based on the *k* values, the adsorption capacity of PMMIPs for BSA was 2.58 times or 3.52 or 4.30 times larger than that for OVA, BHb or Lyz, respectively. The *k*′ values on the bases of OVA, BHb and Lyz were 3.96, 3.74 and 3.77, respectively, both of which were larger than 1. This suggested that PMMIPs had a better adsorption ability for BSA (nearly four times larger) than PMNIPs, owing to the specificity of recognition sites in PMMIPs. These results demonstrated that PMMIPs had good selectivity for the template molecules.

### 3.5. Competitive Adsorption Tests

To further explore the specific recognition of PMMIPs towards BSA, competitive adsorption experiments were implemented by selecting BHb as a competitor. It could be seen from Figure 5b that the adsorption capacity of PMMIPs towards template BSA was higher than that of control BHb. PMMIPs exhibited excellent selectivity for BSA based on an imprinting factor of 2.25 in the presence of interfering protein. 

### 3.6. Removal of BSA from Real Samples

The value of practical application of PMMIPs was also evaluated in a real environment. Milk, urine and sewage were selected as the real samples. As shown in Table S5 (Supplementary Materials), the adsorption capacities of PMMIPs for milk, urine and sewage were 247.22, 245.10 and 247.58 mg g^−1^, respectively, which were slightly lower than 255.26 mg g^−1^: a result obtained under ideal conditions, i.e., in deionized water. These results revealed that approximately 96% of the ideal-state adsorption capacity of PMMIPs for BSA was achieved under complicated conditions. Meanwhile, it could be concluded from Table S6 that the adsorption capacity of PMMIPs towards template BSA was higher than that of control BHb in real samples. This was evidence that PMMIPs have a stable adsorption capacity even in real environments, therefore PMMIPs are a promising candidate for urine analysis in biochemical criterion, and the determination of protein in foods.

### 3.7. Regeneration and Reusability of PMMIPs

**Figure 6:**
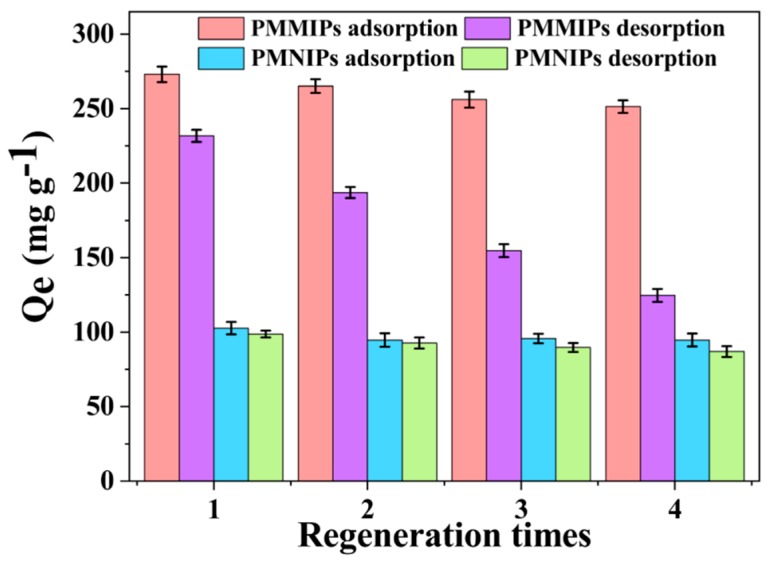
Regeneration and reusability of PMMIPs and PMNIPs for four times. Adsorption conditions: V = 1 mL, m_PMMIPs_ = m_PMNIPs_ = 10 mg, C_BSA_ = 0.2–2.0 mg mL^−1^, T = 20 °C, t = 5 h, pH = 7.0 Desorption conditions: V = 1 mL, m_PMMIPs_ = m_PMNIPs_ = 10 mg, C_BSA_ = 0.2–2.0 mg mL^−1^, T = 35 °C, t = 5 h, pH = 7.0.

Reusability is a vital parameter for the practical application of sorbents. The regeneration capability of the prepared BSA imprinted polymers was investigated via rebinding-desorption cycles by adopting SDS and acetic acid solutions as the eluent. As shown in Figure 6, the adsorption capacity of fresh PMMIPs was 273.07 mg g^-−1^ and the rebinding capacity remained at 90% of the initial rebinding value after four regeneration cycles. The adsorption efficiency loss of PMMIPs for template BSA was less than 10% after four regeneration cycles. Comparing it with some other reported BSA imprinting methods [31,49,50], PMMIPs in this work showed a higher adsorption capacity and a less loss of adsorption capacity after more cycles than the previous study. The possible reason for the decrease in adsorption capacity could be ascribed to the deformation of some recognition sites during repeated washing. Hence, the damaged cavities no longer matched the template BSA spatially. However, the adsorption capacity of PMNIPs was nearly the same as that during the first binding stages. The effect of washing on PMNIPs was negligible on account of the lack of special recognition sites. Theoretically, the desorption capacity should be only slightly less than the corresponding adsorption capacity, in other words, the fact that the change trend of desorption capacity was similar to the one of adsorption capacity. However, the actual result was that the change trend of desorption capacity was dramatically decreased maybe due to some proteins were not completely eluted or the desorbed proteins were not completely detected, this is an urgent problem to be solved in the follow-up study. In summary, the results indicated that the satisfactory regeneration capability of the as-prepared imprinted polymers has potential applications in practice.

## 4. Conclusions

In this work, novel PMMIPs were prepared via a technique that combined precipitation polymerization and surface imprinting for the specific recognition of BSA. The obtained PMMIPs displayed superior adsorption capacity for the template BSA than PMNIPs. The selectivity and specificity of PMMIPs were assessed by tests on the adsorption of the single reference protein, binary protein mixtures, and real samples. The adsorption and desorption of BSA was indirectly regulated by system temperature, which may have benefited from the existence of a temperature-sensitive imprinting layer. The results demonstrated that recognition cavities were formed on the surface of PMMIPs during the imprinting process. Moreover, reusability studies showed that PMMIPs could be easily regenerated with good stability. The simple preparation, facile separation, and favorable selectivity of PMMIPs make it a promising material for the specific recognition of proteins in biomolecular separations.

## Supplementary Materials

The following are available online at https://www.mdpi.com/2073-4360/12/3/536/s1, Figure S1: SEM images of (a) MHNTs@PEG, (b) MHNTs@PEG@4-VP, (c) PMMIPs and (d) PMNIPs; TEM images of (e) MHNTs@PEG, (f) MHNTs@PEG@4-VP, (g) PMMIPs and (h) PMNIPs; Figure S2: DTG plots of (a) MHNTs@PEG, (b) MHNTs@PEG@4-VP and (c) PMMIPs; Table S1: The effects of synthesis conditions of MIPs on the *Q*_e_ of PMMIPs and IF for BSA; Table S2. Isotherm parameters of BSA binding on PMMIPs and PMNIPs by two equilibrium adsorption models; Table S3: Kinetic parameters of BSA binding on PMMIPs and PMNIPs by two rate equations; Table S4: Adsorption selectivity of PMMIPs and PMNIPs; Table S5: Adsorption Properties of PMMIPs in real environment; Table S6: Competitive adsorption tests of BSA and BHb on PMMIPs in real environment.
