# Supplementary material for: PEGylated Thermo-Sensitive Bionic Magnetic Core-Shell Structure Molecularly Imprinted Polymers Based on Halloysite Nanotubes for Specific Adsorption and Separation of Bovine Serum Albumin
Source: Polymers (Basel). 2020 Mar 2;12(3):536. doi: 10.3390/polym12030536 (PMC7182869; doi:10.3390/polym12030536)
Supplement: Supplementary file 1 [file polymers-12-00536-s001.pdf]

Support Information

PEGylated thermo-sensitive bionic magnetic core-shell structure molecularly imprinted polymers based on halloysite nanotubes for specific adsorption and separation of bovine serum albumin

Xiufang Li[1], Hui Liu[1], Zhiwei Deng[1], Wenqing Chen[1], Tianhao Li[1], Yunshan Zhang[1], Zhuomin Zhang[1], Yao He[1], Zhijian Tan[2*] and Shian Zhong[1*]

[1]College of Chemistry and Chemical Engineering, Central South University, Changsha, Hunan 410083, (P.R. China)

[1*]College of Chemistry and Chemical Engineering, Central South University, Changsha, Hunan 410083, (P.R. China) [1*]**E-mail: zhongshian@aliyun.com**

[2*]Institute of Bast Fiber Crops, Chinese Academy of Agricultural Sciences, Changsha, Hunan 410205, (P.R. China) [2*]**E-mail: tanzhijian@caas.cn**

2. Experimental section

2.1. Instruments and apparatus

Infrared spectrum results were acquired using a FT-IR spectrometer (Nicolet, USA), Ultraviolet-visible spectra were tested by using a UV-2450 spectrophotometer (Shimadzu, Japan). The morphologies of prepared materials were acquired by scanning electron microscopy (SEM) using a Nova Nano SEM 230 (FEI Electron Optics). Morphology and size images of the products were acquired at 200kV by using a JEOL 2100F microscope (JEOL, Japan). Thermo-gravimetric analysis (TGA) data from products were obtained under N_2 atmosphere using a TG system (TA Q600 V8.0, USA); temperatures ranging from 25 °C to 850 °C at the heating speed of 10 °C per minute were used. Particle phases were characterized using X-ray diffraction (XRD, Rigaku D/max22500 XRD) with Cu and Ka radiation (Rigaku Ltd, Japan). The magnetic properties of particles were monitored at room temperature using vibrating sample magnetometry (VSM, Lake Shore Ltd). Specific surface area (BET) analysis was performed using specific surface area analyzers (ASAP2020, Micromeritics Instrument Corp, USA).

2.2. Preparation of PEGylated magnetic halloysite nanotubes

PEGylated magnetic halloysite nanotubes (MHNTs@PEG) were synthesized according to previously published literatures with some modifications [1]. Briefly, HNTs were first purified and vacuum dried at 120 °C for 8 h. HNT powder (0.25 g) was added to deionized water (75 mL), followed by ultrasonication for 15 min for dispersion, then addition of $FeCl_3.6H_2O$ (0.65 g, 2.4mmol), $FeCl_2$. $4H_2O$ (0.24 g, 1.2 mmol) and PEG-6000 (1.50 g) (system I). PEG 6000 (0.3 g) was then dissolved into deionized water (2.5 mL), and $NH_3{\cdot}H_2O$ (5 mL) was added (system II). Finally, System I was dispersed and stirred for 10 min at 50 °C under argon. System II was added to System I dropwise using a syringe; a black solid was formed after incubation the reaction for 3 h at 70 °C. After external magnetic field separation, the black solid product was collected, then washed with deionized water until it reached pH 7. The black solid was vacuum dried at 30 °C for 24 h under to acquire MHNTs@PEG (0.4 g).

2.3. Modification of MHNTs@PEG

To obtain 4-vinyl pyridine (4-VP) modified MHNTs@PEG (MHNTs@PEG@4-VPs), the synthesis was carried out in the hydrophobic phase. 4-VP (50 mg) was disappeared into acetonitrile (7.5 mL) by ultrasonic for 20 min. Then, MHNTs@PEG (100 mg) and crosslinking agent EGDMA (200 mg) were dissolve into the above-mentioned diaphanous solution. The solution was then deoxygenated by blowing argon for 20 min under stirring (1000 rpm); then, AIBN (4 mg) was added and vigorously stirring at 55 °C for 3 h to achieve pre-polymerization and at 65 °C for another 24 h. MHNTs@PEG@4-VPs were separated using an external magnet, washed with acetonitrile and ethanol, then dried at 35 °C. The MHNTs@PEG@4-VP product weight was approximately 0.132 g.

3. Results and discussion

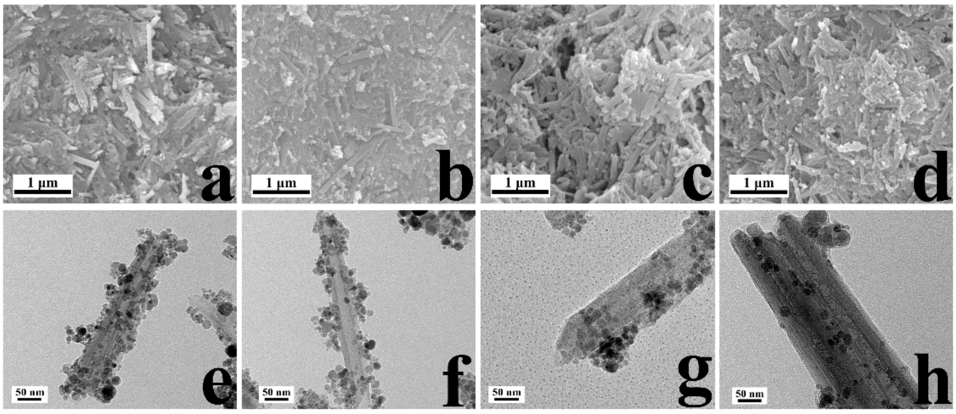

Figure S1. SEM images of (a) MHNTs@PEG, (b) MHNTs@PEG@4-VP, (c) PMMIPs and (d) PMNIPs; TEM images of (e) MHNTs@PEG, (f) MHNTs@PEG@4-VP, (g) PMMIPs and (h) PMNIPs.

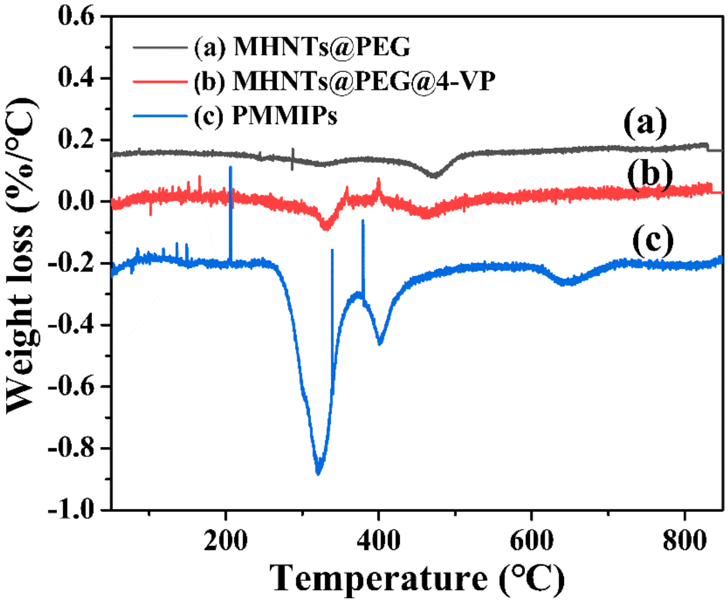

Figure S2. DTG plots of (a) MHNTs@PEG, (b) MHNTs@PEG@4-VP and (c) PMMIPs.

Table S1. The effects of synthesis conditions of MIPs on the Q_e of PMMIPs and *IF* for BSA.

[a]Runs	Template	Functional monomers	Crosslinker	[b]Q_e of PMMIPs (mg g^{-1})	[c]*IF*
	BSA (mg)	NIPAm+AAM+APM (mmol)	BIS (mmol)		
**1**	20	3.6	1.6	49.70	1.58
**2**	30	3.6	1.6	54.60	2.10
**3**	40	3.6	1.6	88.80	2.37
**4**	50	3.6	1.6	113.40	2.49
**5**	60	3.6	1.6	145.60	2.15
**6**	50	1	1.6	76.40	1.98
**7**	50	2	1.6	89.50	2.25
**8**	50	4	1.6	115.37	2.39
**9**	50	6	1.6	108.88	2.21
**10**	50	4	0.5	124.81	2.65
**11**	50	4	1	138.30	2.74
**12**	50	4	2.5	90.90	2.42

[a] All MIPs were obtained by putting the equal amount of MHNTs@PEG@4-VP (200 mg), phosphate buffer (PB) solution (0.1 mol L^{-1}, pH=7.0, 35 mL), APS (100 mg) and TEMED (50 μL) under a argon protection at 40 °C for 24 h, the molar ratios of three monomers nNIPAm/nAAM/nAPM was 1/0.5/0.5.

[b] Q_e was the adsorption capacity of the PMMIPs.

[c] *IF* is the imprinting factor.

Table S2. Isotherm parameters of BSA binding on PMMIPs and PMNIPs by two equilibrium adsorption models.

Adsorbents	Langmuir model			Freundlich model		
	K_L(L/mg)	Q_m(mg/g)	R^2	K_F(mg/g)	1/n	R^2
**PMMIPs**	0.0025	326.42	0.9854	222.7293	0.3530	0.9180
**PMNIPs**	0.0013	156.59	0.9829	85.7205	0.4719	0.9419

Table S3. Kinetic parameters of BSA binding on PMMIPs and PMNIPs by two rate equations.

Adsorbents	$Q_{e,exp}$ (mg g^{-1})[a]	Pseudo-first-order model			Pseudo-second-order model		
		$Q_{e,cal}$ (mg g^{-1})[b]	k_1 (1/min)	R^2	$Q_{e,cal}$ (mg g^{-1})	k_2 (g/(mg•min))	R^2
**PMMIPs**	276.46	270.04	0.07962	0.9764	301.84	0.000339	0.9956
**PMNIPs**	89.83	85.86	0.10567	0.9283	93.54	0.00158	0.9897

[a] $Q_{e,exp}$ (mg g^{-1}) is the experimental value of Q_e.

[b] $Q_{e,cal}$ (mg g^{-1}) is the calculated value of Q_e.

Table S4. Adsorption selectivity of PMMIPs and PMNIPs

Reference compounds	PMMIPs				PMNIPs				k′
	C_e(mg/L)	Q_e	K_d	k	C_e(mg/L)	Q_e	K_d	k	
**BSA**	210.74	243.56	1.16	---	277.53	88.81	0.32	---	---
**OVA**	320.96	145.64	0.45	2.58	214.97	105.34	0.49	0.65	3.96
**BHb**	345.11	113.44	0.33	3.52	267.41	90.92	0.34	0.94	3.74

**Lyz**	365.19	98.60	0.27	4.30	319.64	89.50	0.28	1.14	3.77

Table S5. Adsorption Properties of PMMIPs in real environment

Solvent	Adsorption capacity (mg g^{-1})	Adsorption efficiency loss %
**Deionized water**	255.26 ± 2.13	---
**Milk**	247.22 ± 1.58	3.15
**Urine**	245.10 ± 1.35	3. 98
**Sewage**	247.58 ± 1.74	3.01

Table S6. Competitive adsorption tests of BSA and BHb on PMMIPs in real environment

Solvent	Adsorption capacity BSA (mg/g)	Adsorption capacity BHb (mg/g)
**Deionized water**	242.57 ± 1.32	89.26 ± 2.68
**Milk**	238.15 ± 1.45	82.83 ± 1.76
**Urine**	225.92 ± 1.93	72.17 ± 2.21
**Sewage**	229.10 ± 1.27	75.49 ± 1.59

References

[1] X. Li, J. Chen, H. Lui, Z. Deng, J. B Li, T. Ren, H. Huang, W. Chen, Y. Yang, S. Zhong, β-Cyclodextrin Coated and Folic Acid Conjugated Magnetic Halloysite Nanotubes for Targeting and Isolating of Cancer Cells, Colloids Surf., B:. 181 (2019) 379-388.
